# Crystal structure of (*E*)-5,5-dimethyl-2-[3-(4-nitro­phen­yl)allyl­idene]cyclo­hexane-1,3-dione

**DOI:** 10.1107/S2056989015011172

**Published:** 2015-06-13

**Authors:** Jae Kyun Lee, Sun-Joon Min, Yong Seo Cho, Jang Hyuk Kwon, Junghwan Park

**Affiliations:** a Center for Neuro-Medicine, Korea Institute of Science & Technology, Hwarangro 14-gil, Seongbuk-gu, Seoul 136-791, Republic of Korea; b Department of Biological Chemistry, Korea University of Science and Technology (UST), 176 Gajung-dong, 217 Gajungro, Yuseong-gu, Daejeon 305-333, Republic of Korea; c Department of Information Display, Kyung Hee University, Dongdaemoon-gu, Seoul 130-701, Republic of Korea; d Duksan Neolux Co. Ltd, 21-32, Ssukgol-gil, Ipjang-myeon, Seobuk-gu, Cheonan-si, Chungcheongnam-do 331-821, Republic of Korea

**Keywords:** crystal structure, cyclo­hexane-1,3-dione, dimedone

## Abstract

In the title compound, C_17_H_17_NO_4_, the cylohexane-1,3-dione ring adopts an envelope conformation with the dimethyl-subsituted C atom as the flap. Its mean plane is inclined to the benzene ring by 7.99 (19)°. The mol­ecule has a *trans* conformation about the bridging C=C bonds of the ally­idene chain. In the crystal, mol­ecules are linked *via* pairs of C—H⋯O hydrogen bonds, forming inversion dimers. The dimers are linked by further C—H.·O hydrogen bonds, forming sheets lying parallel to (10-1).

## Related literature   

For the uses of cyclo­hexane-1,3-dione derivatives in various organic synthesis fields, see: Feng *et al.* (2015 ▸); Frolov *et al.* (2013 ▸); Sharma *et al.* (2012 ▸ and references therein).
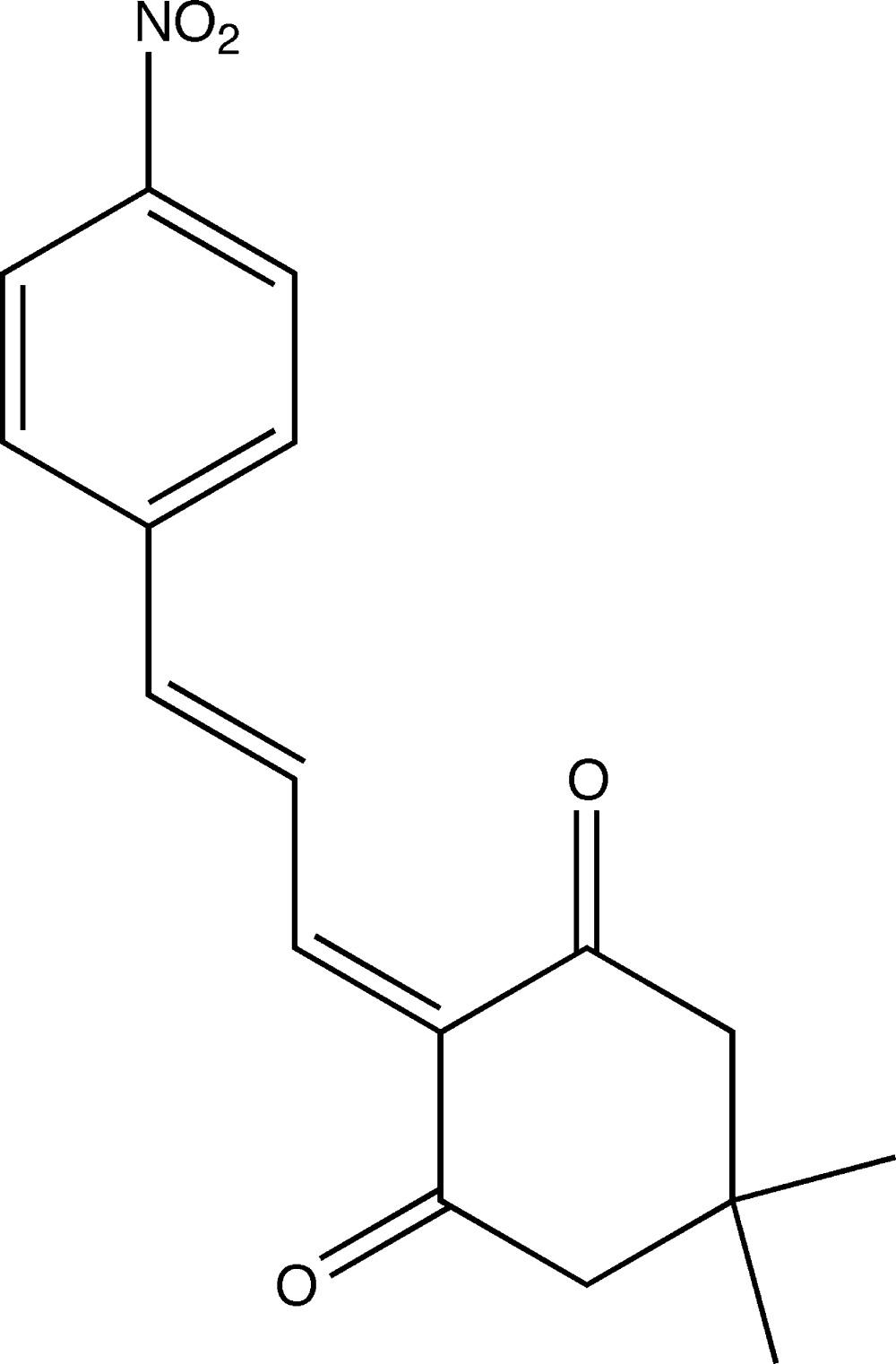


## Experimental   

### Crystal data  

C_17_H_17_NO_4_
*M* *_r_* = 299.33
Monoclinic, 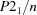

*a* = 13.498 (2) Å
*b* = 7.0791 (9) Å
*c* = 16.1717 (19) Å
β = 91.420 (4)°
*V* = 1544.8 (4) Å^3^
*Z* = 4
Mo *K*α radiation
μ = 0.09 mm^−1^
*T* = 296 K
0.30 × 0.10 × 0.10 mm

### Data collection  

Rigaku R-AXIS RAPID diffractometer
Absorption correction: multi-scan (*ABSCOR*; Rigaku, 1995 ▸) *T*_min_ = 0.641, *T*_max_ = 0.991
14300 measured reflections
3526 independent reflections
1508 reflections with *F* ^2^ > 2.0σ(*F* ^2^)
*R*_int_ = 0.049

### Refinement  

*R*[*F* ^2^ > 2σ(*F* ^2^)] = 0.072
*wR*(*F* ^2^) = 0.258
*S* = 1.04
3526 reflections
213 parameters
H atoms treated by a mixture of independent and constrained refinement
Δρ_max_ = 0.33 e Å^−3^
Δρ_min_ = −0.20 e Å^−3^

### 

Data collection: *RAPID-AUTO* (Rigaku, 2006 ▸); cell refinement: *RAPID-AUTO*; data reduction: *RAPID-AUTO*; program(s) used to solve structure: *Il Milione* (Burla *et al.*, 2007 ▸); program(s) used to refine structure: *SHELXL97* (Sheldrick, 2008 ▸); molecular graphics: *CrystalStructure* (Rigaku, 2010 ▸); software used to prepare material for publication: *CrystalStructure*.

## Supplementary Material

Crystal structure: contains datablock(s) General, I. DOI: 10.1107/S2056989015011172/ff2138sup1.cif

Structure factors: contains datablock(s) I. DOI: 10.1107/S2056989015011172/ff2138Isup2.hkl

Supporting information file. DOI: 10.1107/S2056989015011172/ff2138Isup3.cml

**Fig. 1.**
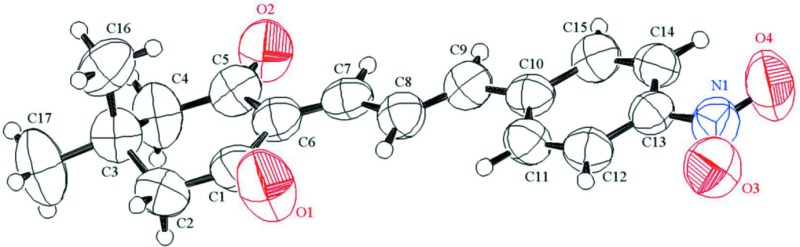
The molecular structure of (I) showing the atomic numbering and 50% probability displacement ellipsoids.

. DOI: 10.1107/S2056989015011172/ff2138fig1.tif
The mol­ecular structure of (I) showing the atomic numbering and 50% probability displacement ellipsoids.

CCDC reference: 1405677

Additional supporting information:  crystallographic information; 3D view; checkCIF report

## Figures and Tables

**Table 1:** Hydrogen-bond geometry (, )

*D*H*A*	*D*H	H*A*	*D* *A*	*D*H*A*
C9H9O2^i^	0.90(3)	2.42(4)	3.305(5)	170(4)
C14H14O4^ii^	0.93	2.52	3.286(4)	140
C16H16*C*O1^iii^	0.96	2.45	3.407(6)	174

Symmetry codes: (i) 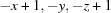; (ii) 
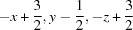
; (iii) 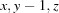.

## supplementary crystallographic information

### S1. Experimental

To solution of 5,5-Dimethyl-1,3-cyclohexanedione (1.84 mmol), 4-nitrocinnamaldehyde(1.84 mmol) and 4 Å MS was added catalytic amounts of *L*-proline in under nitrogen atmosphere. The anhydrous ethyl acetate (2 ml) was added to a reaction mixture and the solution was stirred at room temperature for 3 h. The progress of reaction was monitored by TLC. After completion of reaction, the reaction mixture was filtered through pad of celite to remove MS and evaporation of the solvent afforded a mixture. The mixture was purified by flash column chromatography(EA: Hex = 1: 3) to afford the title compound as a colorless solid in yield 62%. Recrystallization from ethanol gave crystals suitable for X-ray analysis.

### S2. Refinement

All hydrogen atoms were positioned geometrically and refined using a riding model with C—H = 0.93–0.97 Å and *U*iso(H) = 1.2 or 1.5 *U*eq(C).

### Crystal data

**Table:**

C_17_H_17_NO_4_	*F*(000) = 632.00
*M**_r_* = 299.33	*D*_x_ = 1.287 Mg m^−^^3^
Monoclinic, *P*2_1_/*n*	Mo *K*α radiation, λ = 0.71075 Å
Hall symbol: -P 2yn	Cell parameters from 7134 reflections
*a* = 13.498 (2) Å	θ = 3.0–27.5°
*b* = 7.0791 (9) Å	µ = 0.09 mm^−^^1^
*c* = 16.1717 (19) Å	*T* = 296 K
β = 91.420 (4)°	Block, yellow
*V* = 1544.8 (4) Å^3^	0.30 × 0.10 × 0.10 mm
*Z* = 4	

### Data collection

**Table:**

Rigaku R-AXIS RAPID diffractometer	1508 reflections with *F*^2^ > 2.0σ(*F*^2^)
Detector resolution: 10.000 pixels mm^-1^	*R*_int_ = 0.049
ω scans	θ_max_ = 27.5°
Absorption correction: multi-scan (*ABSCOR*; Rigaku, 1995)	*h* = −17→17
*T*_min_ = 0.641, *T*_max_ = 0.991	*k* = −8→9
14300 measured reflections	*l* = −20→20
3526 independent reflections	

### Refinement

**Table:**

Refinement on *F*^2^	Secondary atom site location: difference Fourier map
*R*[*F*^2^ > 2σ(*F*^2^)] = 0.072	Hydrogen site location: inferred from neighbouring sites
*wR*(*F*^2^) = 0.258	H atoms treated by a mixture of independent and constrained refinement
*S* = 1.03	*w* = 1/[σ^2^(*F*_o_^2^) + (0.1342*P*)^2^] where *P* = (*F*_o_^2^ + 2*F*_c_^2^)/3
3526 reflections	(Δ/σ)_max_ < 0.001
213 parameters	Δρ_max_ = 0.33 e Å^−^^3^
0 restraints	Δρ_min_ = −0.20 e Å^−^^3^
Primary atom site location: structure-invariant direct methods	

### Special details

**Table:**

Geometry. All e.s.d.'s (except the e.s.d. in the dihedral angle between two l.s. planes) are estimated using the full covariance matrix. The cell e.s.d.'s are taken into account individually in the estimation of e.s.d.'s in distances, angles and torsion angles; correlations between e.s.d.'s in cell parameters are only used when they are defined by crystal symmetry. An approximate (isotropic) treatment of cell e.s.d.'s is used for estimating e.s.d.'s involving l.s. planes.
Refinement. Refinement was performed using all reflections. The weighted *R*-factor (*wR*) and goodness of fit (*S*) are based on *F*^2^. *R*-factor (gt) are based on *F*. The threshold expression of *F*^2^ > 2.0 σ(*F*^2^) is used only for calculating *R*-factor (gt).

### Fractional atomic coordinates and isotropic or equivalent isotropic displacement parameters (Å^2^)

**Table:**

	*x*	*y*	*z*	*U*_iso_*/*U*_eq_	
O1	0.6218 (3)	0.3243 (5)	0.22209 (16)	0.1482 (15)	
O2	0.4678 (3)	−0.1508 (4)	0.37594 (14)	0.1069 (9)	
O3	0.8119 (3)	1.2273 (4)	0.56257 (19)	0.1160 (10)	
O4	0.7964 (3)	1.1164 (5)	0.68389 (19)	0.1409 (13)	
N1	0.7877 (3)	1.1031 (5)	0.6090 (2)	0.0895 (9)	
C1	0.5711 (3)	0.1820 (5)	0.2258 (2)	0.0874 (11)	
C2	0.5393 (3)	0.0801 (5)	0.1507 (2)	0.0933 (11)	
C3	0.5375 (3)	−0.1341 (5)	0.1603 (2)	0.0867 (10)	
C4	0.4686 (3)	−0.1804 (6)	0.2302 (2)	0.1008 (12)	
C5	0.4923 (3)	−0.0828 (5)	0.3103 (2)	0.0812 (10)	
C6	0.5423 (3)	0.1050 (5)	0.30814 (19)	0.0753 (9)	
C7	0.5588 (3)	0.1923 (5)	0.3802 (3)	0.0736 (9)	
C8	0.6060 (3)	0.3708 (5)	0.3991 (2)	0.0774 (9)	
C9	0.6183 (3)	0.4260 (5)	0.4772 (3)	0.0774 (9)	
C10	0.6637 (3)	0.6006 (4)	0.50901 (18)	0.0713 (8)	
C11	0.7006 (3)	0.7417 (5)	0.45815 (19)	0.0777 (9)	
C12	0.7411 (3)	0.9045 (5)	0.49093 (19)	0.0770 (9)	
C13	0.7450 (3)	0.9282 (4)	0.57532 (18)	0.0714 (8)	
C14	0.7105 (3)	0.7914 (5)	0.62813 (18)	0.0788 (10)	
C15	0.6701 (3)	0.6298 (5)	0.59411 (19)	0.0775 (9)	
C16	0.6418 (3)	−0.2060 (6)	0.1798 (3)	0.1085 (13)	
C17	0.4986 (4)	−0.2254 (7)	0.0804 (3)	0.1313 (18)	
H2A	0.5836	0.1124	0.1065	0.1119*	
H2B	0.4734	0.1228	0.1343	0.1119*	
H4A	0.4015	−0.1473	0.2128	0.1210*	
H4B	0.4702	−0.3157	0.2396	0.1210*	
H11	0.6978	0.7256	0.4011	0.0932*	
H12	0.7656	0.9975	0.4564	0.0924*	
H14	0.7144	0.8079	0.6852	0.0945*	
H15	0.6461	0.5371	0.6291	0.0930*	
H16A	0.6674	−0.1452	0.2289	0.1302*	
H16B	0.6838	−0.1779	0.1343	0.1302*	
H16C	0.6399	−0.3401	0.1884	0.1302*	
H17A	0.4984	−0.3603	0.0867	0.1575*	
H17B	0.5405	−0.1912	0.0357	0.1575*	
H17C	0.4323	−0.1822	0.0685	0.1575*	
H9	0.603 (3)	0.352 (5)	0.520 (2)	0.091 (11)*	
H8	0.630 (3)	0.447 (5)	0.350 (3)	0.113 (12)*	
H7	0.540 (3)	0.130 (4)	0.4304 (18)	0.071 (8)*	

### Atomic displacement parameters (Å^2^)

**Table:**

	*U*^11^	*U*^22^	*U*^33^	*U*^12^	*U*^13^	*U*^23^
O1	0.239 (5)	0.108 (2)	0.0979 (19)	−0.066 (3)	0.012 (2)	0.0151 (15)
O2	0.134 (3)	0.0923 (16)	0.0961 (17)	−0.0244 (15)	0.0325 (16)	0.0069 (13)
O3	0.127 (3)	0.0950 (17)	0.125 (3)	−0.0275 (17)	−0.0065 (18)	−0.0005 (17)
O4	0.193 (4)	0.135 (3)	0.0939 (19)	−0.019 (3)	0.003 (2)	−0.0319 (18)
N1	0.084 (3)	0.095 (3)	0.090 (2)	0.0089 (17)	0.0002 (16)	−0.0124 (18)
C1	0.105 (3)	0.0687 (19)	0.089 (3)	−0.0062 (19)	0.0013 (19)	0.0196 (17)
C2	0.093 (3)	0.103 (3)	0.083 (3)	0.001 (2)	−0.0018 (19)	0.014 (2)
C3	0.079 (3)	0.091 (3)	0.091 (3)	−0.0158 (19)	0.0161 (18)	0.0025 (19)
C4	0.096 (3)	0.114 (3)	0.094 (3)	−0.034 (3)	0.025 (2)	−0.016 (2)
C5	0.067 (3)	0.082 (2)	0.095 (3)	−0.0043 (16)	0.0194 (17)	0.0070 (19)
C6	0.064 (2)	0.0771 (19)	0.085 (2)	0.0068 (15)	0.0053 (16)	0.0045 (17)
C7	0.060 (2)	0.0747 (19)	0.087 (3)	0.0095 (15)	0.0078 (16)	0.0130 (19)
C8	0.075 (3)	0.078 (2)	0.080 (2)	0.0042 (17)	0.0086 (17)	0.0022 (18)
C9	0.075 (3)	0.078 (2)	0.080 (3)	−0.0003 (17)	0.0161 (17)	0.0048 (18)
C10	0.062 (2)	0.0776 (19)	0.0743 (19)	0.0076 (15)	0.0046 (14)	0.0093 (16)
C11	0.083 (3)	0.081 (2)	0.0692 (18)	−0.0030 (18)	0.0011 (16)	0.0062 (16)
C12	0.075 (3)	0.080 (2)	0.0759 (19)	−0.0022 (16)	0.0035 (16)	0.0102 (16)
C13	0.062 (2)	0.0777 (19)	0.0746 (19)	0.0121 (15)	0.0026 (15)	−0.0007 (16)
C14	0.074 (3)	0.098 (3)	0.0643 (17)	0.0220 (18)	0.0083 (15)	0.0040 (17)
C15	0.071 (3)	0.083 (2)	0.079 (2)	0.0034 (17)	0.0201 (16)	0.0067 (17)
C16	0.102 (4)	0.093 (3)	0.133 (4)	0.012 (3)	0.031 (3)	0.004 (3)
C17	0.147 (5)	0.156 (4)	0.092 (3)	−0.065 (4)	0.020 (3)	−0.031 (3)

### Geometric parameters (Å, º)

**Table:**

O1—C1	1.220 (5)	C12—C13	1.375 (5)
O2—C5	1.219 (5)	C13—C14	1.380 (5)
O3—N1	1.207 (5)	C14—C15	1.376 (5)
O4—N1	1.218 (5)	C2—H2A	0.970
N1—C13	1.465 (5)	C2—H2B	0.970
C1—C2	1.467 (5)	C4—H4A	0.970
C1—C6	1.499 (5)	C4—H4B	0.970
C2—C3	1.524 (5)	C7—H7	0.96 (3)
C3—C4	1.517 (5)	C8—H8	1.02 (4)
C3—C16	1.522 (6)	C9—H9	0.90 (4)
C3—C17	1.527 (6)	C11—H11	0.930
C4—C5	1.495 (5)	C12—H12	0.930
C5—C6	1.492 (5)	C14—H14	0.930
C6—C7	1.332 (5)	C15—H15	0.930
C7—C8	1.445 (5)	C16—H16A	0.960
C8—C9	1.327 (5)	C16—H16B	0.960
C9—C10	1.468 (5)	C16—H16C	0.960
C10—C11	1.394 (5)	C17—H17A	0.960
C10—C15	1.392 (5)	C17—H17B	0.960
C11—C12	1.376 (5)	C17—H17C	0.960
			
O1···C3	3.573 (5)	O1···H16A^ii^	2.9432
O1···C7	2.870 (5)	O1···H16B^ii^	3.4608
O1···C8	2.895 (5)	O1···H16C^i^	2.4512
O2···C7	2.722 (4)	O1···H17A^i^	3.5181
O3···C12	2.725 (4)	O2···H11^v^	3.2415
O3···C14	3.549 (5)	O2···H14^iii^	3.5772
O4···C12	3.526 (5)	O2···H15^iv^	3.1368
O4···C14	2.721 (5)	O2···H9^iv^	2.42 (4)
C1···C4	2.916 (6)	O2···H7^iv^	3.14 (3)
C1···C8	3.131 (5)	O3···H2B^xi^	2.6644
C1···C16	3.007 (6)	O3···H15^i^	3.3309
C2···C5	2.910 (5)	O3···H16B^vi^	3.2560
C3···C6	2.928 (5)	O3···H17B^vi^	2.6426
C5···C16	3.082 (6)	O3···H9^i^	3.02 (4)
C6···C16	3.331 (6)	O4···H2B^xi^	3.1389
C8···C11	3.062 (5)	O4···H4A^iii^	3.1931
C10···C13	2.770 (5)	O4···H4B^xii^	3.2774
C11···C14	2.771 (5)	O4···H14^vii^	2.5218
C12···C15	2.750 (5)	O4···H15^vii^	3.1534
O1···C16^i^	3.407 (5)	N1···H2B^xi^	3.1884
O1···C16^ii^	3.535 (6)	N1···H17A^xii^	3.3501
O2···N1^iii^	3.479 (5)	N1···H9^i^	3.35 (4)
O2···C9^iv^	3.305 (5)	C1···H16C^i^	3.5639
O2···C11^v^	3.467 (5)	C2···H17B^xiii^	3.2723
O2···C13^iii^	3.386 (5)	C5···H11^v^	3.3895
O2···C14^iii^	3.502 (5)	C5···H14^iii^	3.4038
O3···C9^i^	3.246 (5)	C6···H14^iii^	3.5247
O3···C10^i^	3.413 (4)	C7···H12^v^	3.3233
O3···C15^i^	3.478 (5)	C7···H15^iii^	3.3646
O3···C17^vi^	3.506 (6)	C8···H12^v^	3.5187
O4···C14^vii^	3.286 (5)	C8···H15^iii^	3.4844
N1···O2^iii^	3.479 (5)	C8···H16B^ii^	2.9222
C5···C14^iii^	3.588 (5)	C9···H16B^ii^	3.3418
C7···C15^iii^	3.371 (5)	C9···H9^iii^	3.37 (4)
C9···O2^iv^	3.305 (5)	C10···H7^iii^	3.51 (3)
C9···O3^v^	3.246 (5)	C11···H2A^ii^	3.2511
C9···C9^iii^	3.459 (5)	C11···H17C^xii^	3.5873
C10···O3^v^	3.413 (4)	C11···H7^i^	3.52 (3)
C11···O2^i^	3.467 (5)	C12···H2A^ii^	3.5420
C13···O2^iii^	3.386 (5)	C12···H17C^xii^	3.2482
C14···O2^iii^	3.502 (5)	C12···H7^i^	3.27 (3)
C14···O4^viii^	3.286 (5)	C13···H17A^xii^	3.4540
C14···C5^iii^	3.588 (5)	C13···H17C^xii^	3.0738
C15···O3^v^	3.478 (5)	C14···H4A^xii^	3.0641
C15···C7^iii^	3.371 (5)	C14···H17C^xii^	3.2608
C16···O1^v^	3.407 (5)	C14···H7^iii^	3.54 (3)
C16···O1^ix^	3.535 (6)	C15···H17C^xii^	3.5933
C17···O3^x^	3.506 (6)	C15···H7^iii^	3.33 (3)
O1···H2A	2.4414	C16···H12^x^	3.3094
O1···H2B	2.8142	C16···H8^ix^	3.32 (4)
O1···H16A	3.3815	C17···H2A^xiii^	3.2929
O1···H8	2.24 (4)	C17···H2B^xiii^	3.5766
O2···H4A	2.7640	C17···H17B^xiii^	3.5288
O2···H4B	2.4958	H2A···C11^ix^	3.2511
O2···H7	2.37 (3)	H2A···C12^ix^	3.5420
O3···H12	2.4360	H2A···C17^xiii^	3.2929
O4···H14	2.4487	H2A···H11^ix^	3.0637
N1···H12	2.5893	H2A···H12^ix^	3.5660
N1···H14	2.6310	H2A···H17B^xiii^	2.8668
C1···H4A	3.2706	H2A···H17C^xiii^	2.8771
C1···H16A	2.6559	H2B···O3^xiv^	2.6644
C1···H16B	3.3322	H2B···O4^xiv^	3.1389
C1···H8	2.84 (4)	H2B···N1^xiv^	3.1884
C1···H7	3.37 (3)	H2B···C17^xiii^	3.5766
C2···H4A	2.6749	H2B···H17B^xiii^	2.7936
C2···H4B	3.2940	H2B···H17C^xiii^	3.5729
C2···H16A	2.6498	H4A···O4^iii^	3.1931
C2···H16B	2.6899	H4A···C14^xv^	3.0641
C2···H16C	3.3200	H4A···H14^iii^	3.3272
C2···H17A	3.3270	H4A···H14^xv^	2.7950
C2···H17B	2.6742	H4B···O1^v^	3.2849
C2···H17C	2.6845	H4B···O4^xv^	3.2774
C4···H2A	3.2949	H4B···H14^xv^	3.5425
C4···H2B	2.6494	H4B···H15^iv^	3.0980
C4···H16A	2.6954	H4B···H8^v^	3.2306
C4···H16B	3.3264	H11···O1^ii^	3.2598
C4···H16C	2.6763	H11···O2^i^	3.2415
C4···H17A	2.6869	H11···C5^i^	3.3895
C4···H17B	3.3157	H11···H2A^ii^	3.0637
C4···H17C	2.6480	H11···H16A^i^	2.9497
C5···H2B	3.2005	H11···H16B^ii^	3.3304
C5···H16A	2.7693	H11···H16C^i^	3.5389
C5···H16C	3.3719	H12···O1^ii^	3.5156
C5···H7	2.53 (3)	H12···C7^i^	3.3233
C6···H2A	3.3212	H12···C8^i^	3.5187
C6···H2B	2.9416	H12···C16^vi^	3.3094
C6···H4A	3.0058	H12···H2A^ii^	3.5660
C6···H4B	3.3153	H12···H16B^vi^	2.8188
C6···H16A	2.7814	H12···H16C^vi^	2.9282
C6···H8	2.77 (4)	H12···H17A^vi^	3.4287
C7···H9	2.58 (4)	H12···H17B^vi^	3.4213
C8···H11	2.8002	H12···H9^i^	3.5055
C9···H11	2.6890	H12···H7^i^	3.1990
C9···H15	2.5987	H14···O2^iii^	3.5772
C9···H7	2.46 (3)	H14···O4^viii^	2.5218
C10···H12	3.2509	H14···C5^iii^	3.4038
C10···H14	3.2619	H14···C6^iii^	3.5247
C10···H8	2.82 (4)	H14···H4A^iii^	3.3272
C11···H15	3.2226	H14···H4A^xii^	2.7950
C11···H9	3.23 (4)	H14···H4B^xii^	3.5425
C11···H8	2.88 (4)	H15···O2^iv^	3.1368
C12···H14	3.2433	H15···O3^v^	3.3309
C13···H11	3.2117	H15···O4^viii^	3.1534
C13···H15	3.2033	H15···C7^iii^	3.3646
C14···H12	3.2406	H15···C8^iii^	3.4844
C15···H11	3.2256	H15···H4B^iv^	3.0980
C15···H9	2.47 (4)	H15···H7^iii^	3.5644
C16···H2A	2.6553	H16A···O1^ix^	2.9432
C16···H2B	3.3223	H16A···H11^v^	2.9497
C16···H4A	3.3261	H16A···H8^v^	3.5313
C16···H4B	2.6477	H16A···H8^ix^	3.1187
C16···H17A	2.6573	H16B···O1^ix^	3.4608
C16···H17B	2.6739	H16B···O3^x^	3.2560
C16···H17C	3.3175	H16B···C8^ix^	2.9222
C17···H2A	2.6813	H16B···C9^ix^	3.3418
C17···H2B	2.6395	H16B···H11^ix^	3.3304
C17···H4A	2.5981	H16B···H12^x^	2.8188
C17···H4B	2.6890	H16B···H8^ix^	2.6763
C17···H16A	3.3181	H16C···O1^v^	2.4512
C17···H16B	2.6487	H16C···C1^v^	3.5639
C17···H16C	2.6802	H16C···H11^v^	3.5389
H2A···H4A	3.5476	H16C···H12^x^	2.9282
H2A···H16A	2.8994	H16C···H8^v^	3.0204
H2A···H16B	2.4946	H17A···O1^v^	3.5181
H2A···H16C	3.5408	H17A···N1^xv^	3.3501
H2A···H17A	3.5499	H17A···C13^xv^	3.4540
H2A···H17B	2.4972	H17A···H12^x^	3.4287
H2A···H17C	2.9722	H17A···H17A^xvi^	3.4319
H2B···H4A	2.5047	H17B···O3^x^	2.6426
H2B···H4B	3.5409	H17B···C2^xiii^	3.2723
H2B···H16A	3.5488	H17B···C17^xiii^	3.5288
H2B···H16B	3.5489	H17B···H2A^xiii^	2.8668
H2B···H17A	3.5235	H17B···H2B^xiii^	2.7936
H2B···H17B	2.8942	H17B···H12^x^	3.4213
H2B···H17C	2.4642	H17B···H17B^xiii^	3.1298
H4A···H16A	3.5919	H17B···H17C^xiii^	3.1616
H4A···H16C	3.5269	H17C···C11^xv^	3.5873
H4A···H17A	2.8775	H17C···C12^xv^	3.2482
H4A···H17B	3.4772	H17C···C13^xv^	3.0738
H4A···H17C	2.3934	H17C···C14^xv^	3.2608
H4B···H16A	2.9308	H17C···C15^xv^	3.5933
H4B···H16B	3.5212	H17C···H2A^xiii^	2.8771
H4B···H16C	2.4608	H17C···H2B^xiii^	3.5729
H4B···H17A	2.5311	H17C···H17B^xiii^	3.1616
H4B···H17B	3.5639	H9···O2^iv^	2.42 (4)
H4B···H17C	2.9556	H9···O3^v^	3.02 (4)
H11···H12	2.3026	H9···N1^v^	3.35 (4)
H11···H9	3.5302	H9···C9^iii^	3.37 (4)
H11···H8	2.3227	H9···H12^v^	3.5055
H14···H15	2.3033	H9···H9^iii^	3.52 (5)
H15···H9	2.2673	H8···C16^ii^	3.32 (4)
H16A···H17A	3.5426	H8···H4B^i^	3.2306
H16A···H17B	3.5402	H8···H16A^i^	3.5313
H16B···H17A	2.9028	H8···H16A^ii^	3.1187
H16B···H17B	2.4779	H8···H16B^ii^	2.6763
H16B···H17C	3.5321	H8···H16C^i^	3.0204
H16C···H17A	2.4938	H7···O2^iv^	3.14 (3)
H16C···H17B	2.9737	H7···C10^iii^	3.51 (3)
H16C···H17C	3.5481	H7···C11^v^	3.52 (3)
H9···H8	2.86 (5)	H7···C12^v^	3.27 (3)
H9···H7	2.28 (5)	H7···C14^iii^	3.54 (3)
H8···H7	2.88 (5)	H7···C15^iii^	3.33 (3)
O1···H4B^i^	3.2849	H7···H12^v^	3.1990
O1···H11^ix^	3.2598	H7···H15^iii^	3.5644
O1···H12^ix^	3.5156	H7···H7^iv^	3.12 (4)
			
O3—N1—O4	122.8 (4)	C3—C2—H2A	108.706
O3—N1—C13	119.6 (3)	C3—C2—H2B	108.699
O4—N1—C13	117.6 (3)	H2A—C2—H2B	107.619
O1—C1—C2	121.2 (4)	C3—C4—H4A	108.507
O1—C1—C6	120.1 (3)	C3—C4—H4B	108.511
C2—C1—C6	118.6 (3)	C5—C4—H4A	108.502
C1—C2—C3	114.2 (3)	C5—C4—H4B	108.499
C2—C3—C4	107.6 (3)	H4A—C4—H4B	107.508
C2—C3—C16	109.7 (3)	C6—C7—H7	118.8 (17)
C2—C3—C17	110.0 (3)	C8—C7—H7	110.2 (17)
C4—C3—C16	110.8 (3)	C7—C8—H8	116 (2)
C4—C3—C17	109.6 (4)	C9—C8—H8	124 (2)
C16—C3—C17	109.3 (4)	C8—C9—H9	122 (2)
C3—C4—C5	115.1 (4)	C10—C9—H9	109 (2)
O2—C5—C4	121.0 (3)	C10—C11—H11	119.427
O2—C5—C6	120.3 (3)	C12—C11—H11	119.430
C4—C5—C6	118.6 (3)	C11—C12—H12	120.346
C1—C6—C5	118.1 (3)	C13—C12—H12	120.327
C1—C6—C7	124.5 (3)	C13—C14—H14	120.902
C5—C6—C7	117.3 (3)	C15—C14—H14	120.900
C6—C7—C8	130.9 (4)	C10—C15—H15	118.901
C7—C8—C9	120.2 (4)	C14—C15—H15	118.915
C8—C9—C10	128.5 (4)	C3—C16—H16A	109.477
C9—C10—C11	123.3 (3)	C3—C16—H16B	109.472
C9—C10—C15	119.1 (3)	C3—C16—H16C	109.474
C11—C10—C15	117.6 (3)	H16A—C16—H16B	109.467
C10—C11—C12	121.1 (3)	H16A—C16—H16C	109.468
C11—C12—C13	119.3 (3)	H16B—C16—H16C	109.469
N1—C13—C12	118.5 (3)	C3—C17—H17A	109.478
N1—C13—C14	119.9 (3)	C3—C17—H17B	109.481
C12—C13—C14	121.6 (3)	C3—C17—H17C	109.471
C13—C14—C15	118.2 (3)	H17A—C17—H17B	109.468
C10—C15—C14	122.2 (3)	H17A—C17—H17C	109.466
C1—C2—H2A	108.706	H17B—C17—H17C	109.463
C1—C2—H2B	108.698		
			
O3—N1—C13—C12	5.2 (5)	O2—C5—C6—C7	0.2 (5)
O3—N1—C13—C14	−175.2 (3)	C4—C5—C6—C1	−3.1 (5)
O4—N1—C13—C12	−175.4 (3)	C4—C5—C6—C7	177.2 (3)
O4—N1—C13—C14	4.2 (5)	C1—C6—C7—C8	−0.7 (6)
O1—C1—C2—C3	143.1 (4)	C5—C6—C7—C8	179.0 (3)
O1—C1—C6—C5	−171.8 (4)	C6—C7—C8—C9	−176.8 (3)
O1—C1—C6—C7	7.9 (6)	C7—C8—C9—C10	−179.8 (3)
C2—C1—C6—C5	6.6 (5)	C8—C9—C10—C11	1.3 (6)
C2—C1—C6—C7	−173.7 (3)	C8—C9—C10—C15	−179.4 (3)
C6—C1—C2—C3	−35.2 (5)	C9—C10—C11—C12	178.9 (3)
C1—C2—C3—C4	57.9 (4)	C9—C10—C15—C14	−179.1 (3)
C1—C2—C3—C16	−62.6 (4)	C11—C10—C15—C14	0.3 (5)
C1—C2—C3—C17	177.2 (3)	C15—C10—C11—C12	−0.4 (5)
C2—C3—C4—C5	−54.4 (4)	C10—C11—C12—C13	−0.1 (5)
C16—C3—C4—C5	65.5 (4)	C11—C12—C13—N1	−179.5 (3)
C17—C3—C4—C5	−173.9 (3)	C11—C12—C13—C14	0.9 (5)
C3—C4—C5—O2	−154.5 (3)	N1—C13—C14—C15	179.4 (3)
C3—C4—C5—C6	28.5 (5)	C12—C13—C14—C15	−1.0 (5)
O2—C5—C6—C1	179.9 (3)	C13—C14—C15—C10	0.4 (5)

Symmetry codes: (i) *x*, *y*+1, *z*; (ii) −*x*+3/2, *y*+1/2, −*z*+1/2; (iii) −*x*+1, −*y*+1, −*z*+1; (iv) −*x*+1, −*y*, −*z*+1; (v) *x*, *y*−1, *z*; (vi) −*x*+3/2, *y*+3/2, −*z*+1/2; (vii) −*x*+3/2, *y*+1/2, −*z*+3/2; (viii) −*x*+3/2, *y*−1/2, −*z*+3/2; (ix) −*x*+3/2, *y*−1/2, −*z*+1/2; (x) −*x*+3/2, *y*−3/2, −*z*+1/2; (xi) *x*+1/2, −*y*+3/2, *z*+1/2; (xii) *x*+1/2, −*y*+1/2, *z*+1/2; (xiii) −*x*+1, −*y*, −*z*; (xiv) *x*−1/2, −*y*+3/2, *z*−1/2; (xv) *x*−1/2, −*y*+1/2, *z*−1/2; (xvi) −*x*+1, −*y*−1, −*z*.

### Hydrogen-bond geometry (Å, º)

**Table:**

*D*—H···*A*	*D*—H	H···*A*	*D*···*A*	*D*—H···*A*
C9—H9···O2^iv^	0.90 (3)	2.42 (4)	3.305 (5)	170 (4)
C14—H14···O4^viii^	0.93	2.52	3.286 (4)	140
C16—H16*C*···O1^v^	0.96	2.45	3.407 (6)	174

Symmetry codes: (iv) −*x*+1, −*y*, −*z*+1; (v) *x*, *y*−1, *z*; (viii) −*x*+3/2, *y*−1/2, −*z*+3/2.
