# Supplementary figures and images for: Crystal structure of (E)-5,5-dimethyl-2-[3-(4-nitro­phen­yl)allyl­idene]cyclo­hexane-1,3-dione
Source: Acta Crystallogr E Crystallogr Commun. 2015 Jun 13;71(Pt 7):o485–6. doi: 10.1107/S2056989015011172 (PMC4518916; doi:10.1107/S2056989015011172)

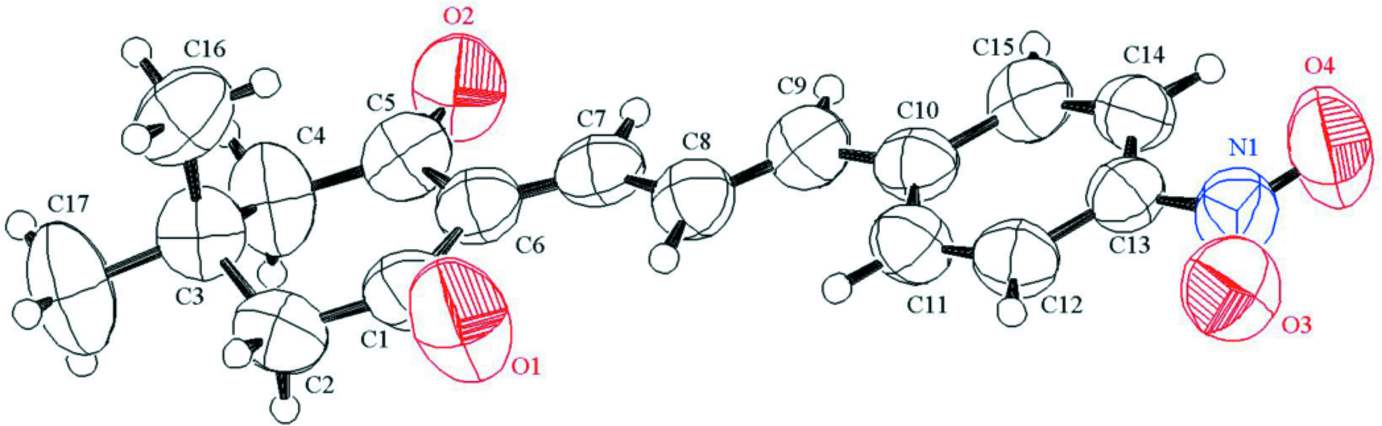

Supplement: Supplementary file 4 [file e-71-0o485-fig1.tif]
